# 3-Hydroxybutyrate Is Active Compound in Flax that Upregulates Genes Involved in DNA Methylation

**DOI:** 10.3390/ijms21082887

**Published:** 2020-04-21

**Authors:** Justyna Mierziak, Wioleta Wojtasik, Anna Kulma, Mariusz Dziadas, Kamil Kostyn, Lucyna Dymińska, Jerzy Hanuza, Magdalena Żuk, Jan Szopa

**Affiliations:** 1 Department of Genetic Biochemistry, Faculty of Biotechnology, Wroclaw University, Przybyszewskiego 63, 51-148 Wroclaw, Poland; justyna.mierziak@uwr.edu.pl (J.M.); anna.kulma@uwr.edu.pl (A.K.); magdalena.zuk@uwr.edu.pl (M.Ż.); szopa@ibmb.uni.wroc.pl (J.S.); 2 Faculty of Chemistry, University of Wroclaw, 50-353 Wroclaw, Poland; mariusz.dziadas@uwr.edu.pl; 3 Department of Genetics, Plant Breeding and Seed Production, Faculty of Life Sciences and Technology, Wroclaw University of Environmental and Plant Sciences, Plac Grunwaldzki 24A, 53-363 Wroclaw, Poland; kamilkostyn@o2.pl; 4 Department of Bioorganic Chemistry, Wroclaw University of Economics and Business, Komandorska 118/120, 53-345 Wroclaw, Poland; lucyna.dyminska@ue.wroc.pl; 5 Institute of Low Temperature and Structure Research, Polish Academy of Sciences, P.O. Box 1410, 50-950 Wroclaw, Poland; j.hanuza@intibs.pl

**Keywords:** 3-hydroxybutyrate, β-ketothiolase, methyltransferase 3, DNA methylation, lignin metabolism, flax

## Abstract

In mammalian cells, 3-hydroxybutyrate (3-HB) is not only an intermediate metabolite during the oxidation of fatty acids, but also an important signaling molecule. On the other hand, the information about the metabolism or function of this compound in plants is scarce. In our study, we show for the first time that this compound naturally occurs in flax. The expression of bacterial *β-ketothiolase* in flax affects expression of endogenous genes of the 3-HB biosynthesis pathway and the compound content. The increase in 3-HB content in transgenic plants or after control plants treatment with 3-HB resulted in upregulation of genes involved in chromatin remodeling. The observation that 3-HB is an endogenous activator of *methyltransferase 3* (*CMT3*), *decreased DNA methylation I* (*DDM1*), *DEMETER DNA glycosylase* (*DME*), and an inhibitor of *sirtuin 1* (*SRT1*) provides an example of integration of different genes in chromatin remodeling. The changes in chromatin remodeling gene expression concomitant with those involved in phenolics and the lignin biosynthesis pathway suggest potential integration of secondary metabolic status with epigenetic changes.

## 1. Introduction

The search for potential plant-derived polymers to act as a sustainable replacement for petrochemicals is a great challenge for agrobiotechnology. A good example is the use of hydroxylated fatty acids as building blocks for the synthesis of poly-3-hydroxyalkanoates. Several bacteria accumulate 3-hydroxybutyrate (3-HB) polymer polyhydroxybutyrate (PHB), which can serve carbon storage and, in consequence, energy source, especially under stressful or limited-nutrient conditions [1,2,3]. Whilst the biosynthesis process of PHB in bacteria is well understood, the mechanism and products of the intracellular PHB depolymerization are still not fully elucidated. Bacteria mobilize PHB granules through the hydrolytic activity of the depolymerase (PhaZ) under certain environmental and nutritional conditions [4]. Some factors, such as guanosine tetraphosphate level, protein synthesis rate, cellular energy demand, and oxidative stress, are suggested as possible triggers for PHB mobilization [5]. Initially, investigated degradation assays carried out with isolated PHB granules revealed, as a first step, the release of 3-hydroxybutyrate (3-HB) in a hydrolytic reaction, then conversion to 3-hydroxybutyryl coenzyme A and further to acetyl-CoA via acetoacetyl-CoA in the reverse direction and by the same enzymes of the synthesis pathway. In a more recent study, the intracellular degradation of PHB by PHB depolymerase PhaZ1 to (n)-3-HB-CoA and its conversion to crotonyl-CoA converted to acetoacetyl-CoA finally cleaved by a 3-ketoacyl–CoA thiolase to two molecules of acetyl-CoA was shown [6,7].

In vertebrates, 3-HB is a product of the normal metabolism of fatty acid oxidation. However the possibility that part of 3-HB derives from bacterial PHB metabolism cannot be excluded as gut microbiota can be a source of PHB [8]. The possibility that part of 3-HB derives from bacterial PHB metabolism cannot be excluded. The 3-HB in humans serves as a metabolic fuel in tissues and its synthesis is increased during metabolic stress conditions, such as exercise, fasting, and severe acute illness. Furthermore, 3-HB has been reported to lower reactive oxygen species (ROS) dependent oxidative stress [9], to inhibit histone deacetylase (HDAC) induced by oxidative stress [10] and to promote the expression of brain-derived neurotrophic factor [11]. It has also been reported that specific supplementation with 3-HB increases lifespan in *C*. *elegans* roundworms by 20% [12]. A more recent study reported that 3-HB infusion in ischemia in mice reduced infarct size, mediated by histone deacetylase sirtuin 3, and reduced ROS production [13]. In the mitochondria 3-HB is also known to increase the expression of neurotrophic factor that facilitates respiration [14]. Very recently the study of healthy human subjects with and without continuous infusion of 3-HB demonstrated reduced cerebral glucose uptake and neuroprotective effects [15]. There is a striking similarity between the activity of polymer in bacteria and monomeric 3-HB in eukaryotic cells. This might suggest that the role of 3-HB is not only as metabolic fuel but probably also as a signaling molecule regulating global processes presumably by chromatin remodeling.

In plants, PHB is mostly known as a product of transformation with bacterial genes. Direct production of PHB in crop plants is an advantageous route for large-scale manufacture of this polymer, especially in energy crops, where a plant byproduct (biomass, seed oil, seedcake) could be used for the production of energy [16]. The polymer of high importance for industry is a substitute for plastics derived from petrochemistry due to similar mechanical properties. Much is known about converting PHB to a range of chemical intermediates. For example, it can be thermally converted to crotonic acid, which can be readily transformed to a number of chemicals including propylene [17] and butanol [18].

Initially questionable, reports have shown that PHB is naturally synthesized in non-transgenic plants. During a study of PHB synthesis in transgenic flax, we also detected PHB in a non-transgenic plant [19] and deeper studies clearly demonstrated PHB biosynthesis in rice from acetate [20]. While the PHB biosynthesis process in a plant can be regarded as understandable, the explanation of the mechanism of the intracellular PHB depolymerization reaction is completely ignored. The knowledge about the physiological significance of PHB in plants is scarce. Few reports concern this and rather concentrate on the role of bacteria produced PHB in plant physiology. For example, one report suggested that for the nitrogen fixation process between leguminous plants and *Rhizobium* the energy required is derived from PHB metabolism. The products transported to the nodules support nitrogen fixation by providing carbon skeletons [21]. Recently, another report suggested that both PHB and glycogen, the major carbon storage compounds in *Sinorhizobium meliloti*, play an important role in rhizobia–legume symbiosis. It was shown that the ability to synthesize PHB is important for atmospheric nitrogen fixation in *Medicago truncatula* nodules and younger *Medicago sativa* nodules. However, the blocking of glycogen synthesis resulted in lower levels of nitrogen fixation on *M. truncatula* and older nodules on *M. sativa*. The data thus suggest that both PHB and glycogen function in the interactions of *S. meliloti* with *Medicago* spp. It should be pointed out that the bacteria strains unable to synthesize PHB or glycogen were still able to form nodules and fix nitrogen, however molecular mechanism needs to be elucidated [22].

Another report evidenced that the deletion of genes involved in the synthesis and degradation of PHB in plant growth-promoting bacteria reduced the ability of the bacteria to enhance plant growth but with little effect on overall root colonization. The data suggest that PHB metabolism likely plays an important role in supporting specific metabolic routes utilized by the bacteria to stimulate plant growth [23].

To summarize, biosynthesis and biodegradation of PHB by different symbiotic and infectious plant bacteria as well as the physiological role of the biopolymer in bacterial cells are well evidenced. However, in plants, there is scant knowledge on both 3-HB and PHB metabolism. It is as yet not known if the physiological effect derives from PHB as an energy source and/or from its degradation products (e.g. 3-HB) acting as signal molecules.

The aim of this study was firstly to identify 3-HB in plants, secondly to analyze expression of the key genes involved in its synthesis, and thirdly to determine its effect on selected flax transcripts by enhancing its synthesis via transforming plants with the bacterial *β-ketothiolase* gene and by treatment of plants with the exogenous 3-HB standard. The focus was on the participation of 3-HB in the regulation of genes involved in the processes of chromatin modification (histone deacetylases and acetylases, methyltransferases) and structural genes involved in the regulation of secondary metabolism (selected genes of the phenylpropanoid pathway).

## 2. Results

### 2.1. Transgenic Flax Plant Selection and Analysis

The selection of flax plants after *Agrobacterium* transformation was performed on a DNA template using primers detecting the presence of a fragment of the genetic construct beginning in the promoter sequence and ending in the sequence of the *phbA* gene. After initial selection, three lines were chosen for further analysis: H1, H5, H15, in which the presence of the gene construct was demonstrated.

**Figure 1:**
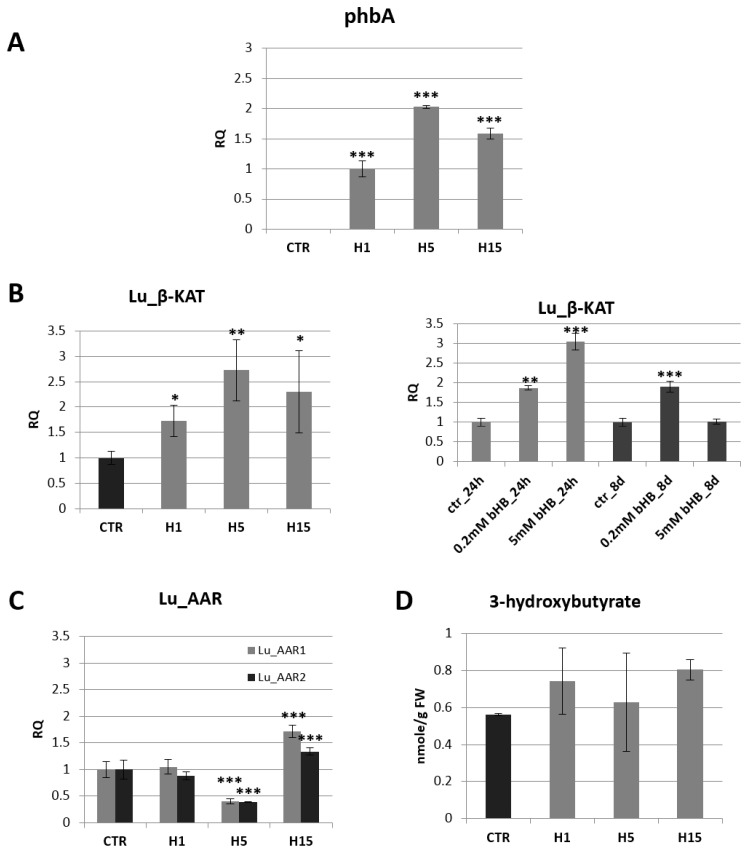
The mRNA level of bacterial beta-ketothiolase (phbA) in transgenic flax plants (H1, H5, H15). Changes in mRNA levels are shown as the relative quantity (RQ) relative to the reference gene (*actin*) in comparison with the expression in H1 plants, where it was the lowest. Non-transgenic control lines (CTR) showed not *phbA* gene expression. The results were obtained via PCR on cDNA, subsequent electrophoretic separation of obtained products and the densitometric measurement of bands (**A**). The mRNA level of endogenous beta-ketothiolase in transgenic flax lines H1, H5, H15 in comparison with non-transgenic flax (CTR) and in flax treated with the beta-hydroxybutyrate standard at concentrations of 0.2 mM and 5 mM during 24 h and 8 days in relation to non-treated flax (CTR) (**B**). The mRNA level of acetoacetyl-CoA reductases in transgenic flax lines H1, H5, H15 in relation to non-transgenic flax (CTR). The mRNA level of Lu_AAR1, Lu_AAR2 (acetoacetyl-CoA reductases) in transgenic flax in comparison with non-transgenic flax (CTR) was obtained from real-time RT-PCR analysis. *Actin* was used as a reference gene and the transcript levels were normalized to those of the control plant (CTR). RQ: relative quantity (**C**). Content of beta-hydroxybutyrate in 5-week-old plants (stems and leaves) from in vitro culture of transgenic flax lines H1, H5, H15 and in non-transgenic flax (CTR) determined using the GC-MS technique (**D**). The results are presented as mean values of three independent repeats ± SD. Statistically significant differences are marked with asterisks (*—*p* < 0.05, **—*p* < 0.01, ***—*p* < 0.001).

In the next step, the mRNA level of the introduced gene (*phbA*) was examined by semi-quantitative PCR (Figure 1A). All tested transgenic lines showed the presence of mRNA of the introduced gene coding for bacterial β-ketothiolase. The highest level of mRNA was observed in the H5 line and it was twice as high as in the H1 line, which showed the lowest level of phbA mRNA.

The analysis of the mRNA level of endogenous beta-ketothiolase showed a significant increase in transgenic lines compared to control plants (Figure 1B). This level was higher by 73%, 173%, 131% compared to the control (for lines H1, H5, H15, respectively). An increase in endogenous gene expression was also observed in wild-type flax treated with exogenous beta-hydroxybutyrate (Figure 1B). After 24 h in flax treated with a lower concentration of 3-HB (0.2 mM), a 1.9-fold increase in the level of mRNA of β-ketothiolase was observed, and in flax treated with a higher concentration of 3-HB (5 mM) a 3.1-fold increase was noted in comparison with untreated plants. After 8 days, in flax treated with 0.2 mM 3-HB, the increase in mRNA level remained at the same level, while in plants treated with 5 mM 3-HB, this level decreased to a level similar to that observed in untreated control plants.

In the bacterial pathway of 3-HB synthesis, in addition to β-KAT, there is another enzyme, acetoacetyl-CoA reductase (AAR). In silico analysis showed the presence of two gene sequences corresponding to *acetoacetyl-CoA reductase*, which was designated *Lu_AAR1* GenBank: AFSQ01023998.1, item 18783–20354) and *Lu_AAR2* (GenBank: AFSQ01003686.1, item 15485–17097). The level of Lu_AAR1 and Lu_AAR2 mRNA was different in all transgenic flax lines (Figure 1C). The transgenic flax line H5 showed more than a twofold decrease in the mRNA level of both acetoacetyl-CoA reductases. The transgenic flax line H15 showed an increase by 72% and 34% for Lu_AAR1 and Lu_AAR2, respectively. The H1 line showed no significant change in mRNA levels for acetoacetyl-CoA reductase as compared to non-transgenic controls.

### 2.2. Determination of 3-Hydroxybutyrate Content in Flax

**Figure 2:**
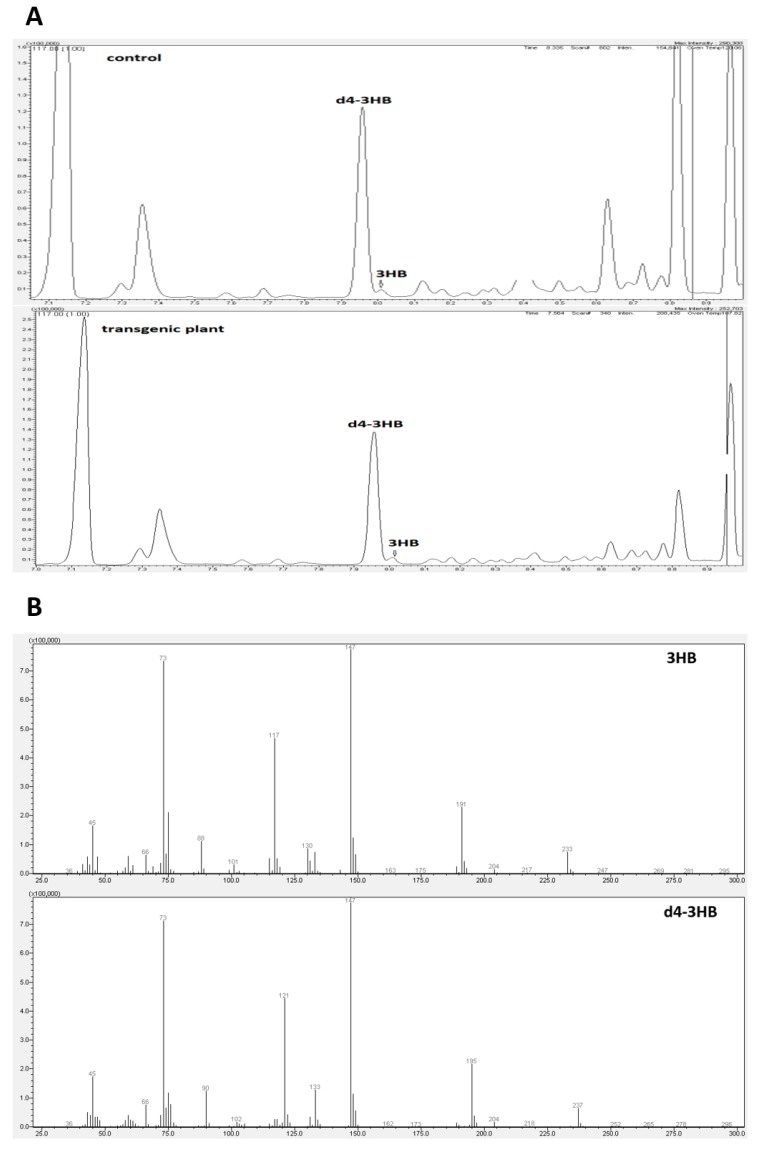
Composition analysis of extracted material from control and transgenic flax with the identification of 3-hydroxybutyrate (3-HB) by GC-MS. (**A**): Counts vs. acquisition time (min) for control (upper panel) and transgene (down panel). (**B**): Counts vs. mass charge ratio (m/z) for 3-HB (upper panel) and deuterated 3-HB (down panel).

There is no accurate information in the literature on the synthesis and function of beta-hydroxybutyrate in plants. Our research was the first to show that this compound occurs naturally in flax. Gas chromatography (Figure 2A,B) and mass spectrometry techniques were used to identify and determine this compound.

3-hydroxybutyrate and its deuterated derivative served as a positive control. To confirm the identity of the 3-HB, the mass spectrometry was used. Two chromatogram peaks with a retention time of 7.96 min and 8.01 min were compared with the data from the mass spectrum library. The results showed that the two peaks matched those of 3-hydroxybutyrate (3-HB) and its deuterated derivative (Figure 2). It can be concluded that the material was composed of 3-HB. The full MS of β-hydroxybutyric acid, 2TMS derivative (C_10_H_24_O_3_Si_2_), showed a parental ion with a m/z 248, which corresponded to the expected mass of 3-HB (NIST14 Mass Spectrometry Data Center Collection, 2014). Fragmentation of this ion led to four main daughter ions, m/z 73, 87, 117 and 147 (Figure 2, thereby confirming the identity of 3-HB (NIST14 Mass Spectrometry Data Center Collection, 2014)).

The content of 3-hydroxybutyrate in 5-week-old green tissue (stems and leaves) of non-transgenic flax was 0.56 nmol/g FW. The transgenic flax line H15 was characterized by the highest 3-HB content among the analyzed transgenic flax plants and contained 0.81 nmol/g FW, which was 43.2% more than in the non-transgenic plants. Other lines contained more of it by 32.4% and 11.9% (lines H1 and H5, respectively) (Figure 1D).

**Figure 3:**
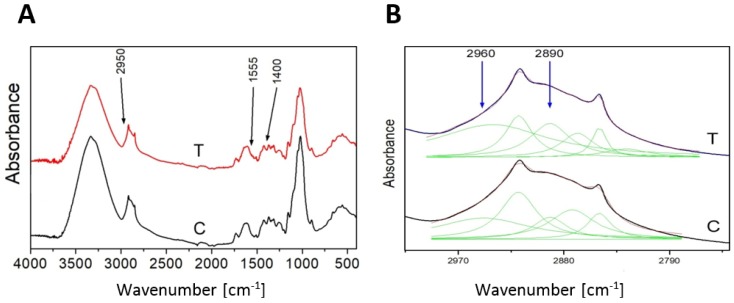
FT-IR spectrum of control (C) and transgenic sample (T) as an example (**A**). The deconvolution of the contour 2800–3000 cm^−1^ into the Lorentz components (**B**).

As additional confirmation of the 3-HB presence in flax the FT-IR analysis was performed. The analyzed spectra reveal the presence of characteristic bands for 3-HB [24]. The contour in the range 2800–3000 cm^−1^ is assigned to C-H stretching vibrations (Figure 3A). The deconvolution of the contour into the Lorentz components showed five peaks, and two of them, 2890 cm^−1^ and 2960 cm^−1^, are attributed to the C-H stretching vibrations of the -CH2-CH2–CH2- group [25] of the 3-hydroxybutyrate (Figure 3B). The amount of absorption of both peaks for the transgenic plant is higher than for the control, which confirms higher levels of 3-HB in transgenic plants. 

### 2.3. Analysis of mRNA Levels of Genes Involved in Epigenetic Modifications

**Figure 4:**
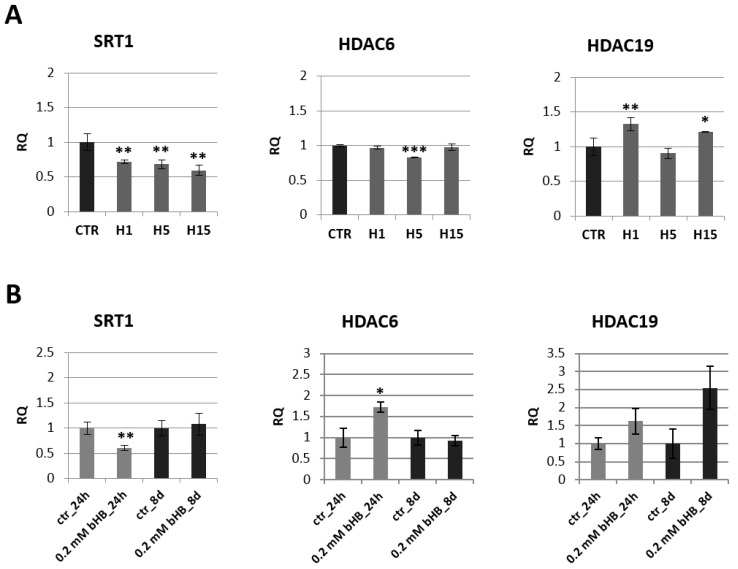
Expression of histone deacetylases in transgenic flax plants (**A**) and in flax treated with 3-HB (**B**). The mRNA level of sirtuin 1 (SRT1) and other histone deacetylases (HDAC6, HDAC19) in transgenic flax or in plants treated with the beta-hydroxybutyrate standard at concentrations of 0.2 mM during 24 h and 8 days in comparison with control (CTR) was obtained from the real-time RT-PCR analysis. *Actin* was used as a reference gene and the transcript levels were normalized to those of the control plant. RQ: relative quantity. The results are presented as mean values of three independent repeats ± SD. Statistically significant differences between the means were marked with asterisks (*—*p* < 0.05, **—*p* <0.01, ***—*p* < 0.001).

Due to literature reports stating that 3-HB may be the regulatory molecule of many signaling pathways in animal cells, levels of gene transcripts involved in epigenetic modifications in the transgenic and non-transgenic flax plants, as well as in plants treated with the 3-HB standard, were evaluated (Figure 4A,B, Figure S1).

A decrease of mRNA level of histone deacetylase SRT1 was observed in all transgenic flax lines compared to control plants. The largest decrease by 40.6% was recorded for the line H15. The other two lines contained a lower SRT1 transcript level by 28.0% (line H1) and 31.5% (line H5) relative to non-transgenic plants. In flax treated with the 3-HB standard, after 24 h, we noted a two-fold decrease in the mRNA level of this gene, and after 8 days the level of SRT1 mRNA in the treated plants was close to the level of control plants. After 24 h in flax treated with the 3-HB standard, their HDAC6 mRNA increased by 72.8% compared to the control. In transgenic plants, no changes in mRNA level of this histone deacetylase were observed, except for a slight decrease in line H5. An increase in HDAC19 mRNA level was observed in lines H1 and H15, while no changes were observed in flax treated with 3-HB.

According to literature data, 3-HB is an inhibitor of histone deacetylases in animals. Our research on human fibroblasts in in vitro culture confirmed that 3-HB significantly reduces also the mRNA level of histone deacetylases (Figure S2) and not only as is described in literature influences enzyme activity.

Histone acetyltransferases are opposing enzymes for histone deacetylases. No significant changes in mRNA levels of genes encoding these enzymes were observed in transgenic plants compared to controls (Figure S1).

**Figure 5:**
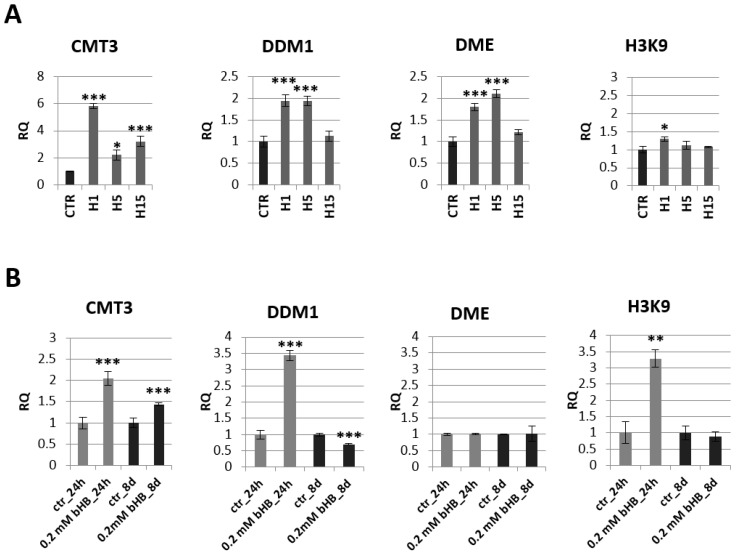
Expression profile of genes encoding enzymes involved in epigenetic modifications in transgenic plants lines H1, H5, H15 and control plants (CTR) (**A**) and in flax treated with the beta-hydroxybutyrate standard at concentration of 0.2 mM during 24 h and 8 days and control plants (CTR) (**B**). The mRNA level of CMT3 (chromomethylase 3), DDM1 (decreased DNA methylation I), DME (glycosylase: DNA demethylase DEMETER) H3K9 (histone methyltransferase H3K9) in transgenic flax or in plants treated with the beta-hydroxybutyrate standard in comparison with control plants (CTR) was obtained from the real-time RT-PCR analysis. *Actin* was used as a reference gene and the transcript levels were normalized to those of the control plant (CTR). RQ: relative quantity. The results are presented as mean values of three independent repeats ± SD. Statistically significant differences between the means were marked with asterisks (*—*p* < 0.05, **—*p* < 0.01, ***—*p* < 0.001).

The analysis of the expression of genes involved in epigenetic modifications in transgenic plants indicated the greatest changes in the chromomethylase 3 (CMT3) mRNA level (Figure 5A). Line H1 showed a nearly 6-fold increase in CMT3 mRNA level compared to the control, line H5 2.2-fold and line H15 3.2-fold. Lines H1 and H5 also showed an approx. 2-fold increase in mRNA level for the gene encoding DDM1 (decreased DNA methylation I) and DME (glycosylase: DNA demethylase DEMETER) (Figure 4A). No significant changes in H3K9 (histone methyltransferase H3K9) mRNA levels were observed in the transgenic plants (except for a slight increase in the H1 line).

The mRNA level of these genes was also analyzed in plants treated with the 3-HB standard (Figure 5B). Similar to transgenic plants overexpressing *beta-ketothiolase*, flax exogenously treated with 3-HB showed an increase in mRNA levels of CMT-3 at both tested times (by 100% and 50%, respectively). Flax treated with exogenous 3-HB showed a 3.5-fold increase in DDM1 level after 24 h, but this DDM1 mRNA level dropped below the level observed in control plants after 8 days. After 24 h of treatment with 3-HB, a 3.3-fold increase in H3K9 mRNA was observed. No change in DME mRNA level was noted in plants treated with 3-HB. 

In addition to analyzing the level of mRNA of genes involved in the process of histone modification and DNA methylation, the level of global DNA methylation in transgenic flax lines was also measured but it showed no significant changes compared to control plants (data not shown).

### 2.4. Analysis of mRNA Levels of Genes Involved in the Phenylpropanoid Pathway and the Content of Selected Metabolites of This Pathway

**Figure 6:**
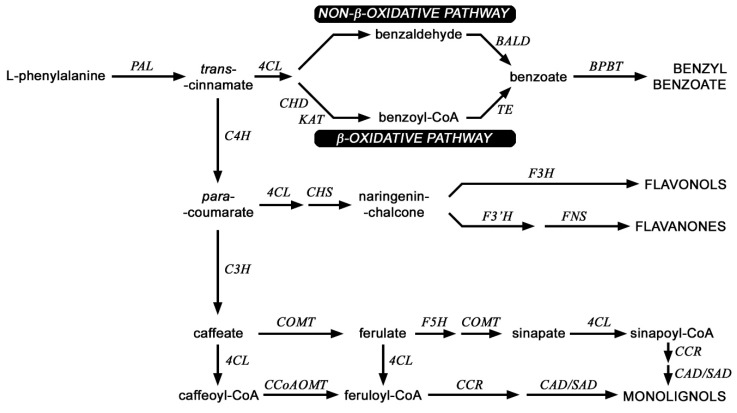
Scheme of selected routes of phenylpropanoid pathway in flax. PAL (phenylalanine ammonia lyase), C4H (cinnamic acid 4-hydroxylase), C3H (4-coumarate 3-hydroxylase), CCR (cinnamoyl-CoA reductase), F5H (ferulate 5-hydroxylase), SAD (sinapyl alcohol dehydrogenase), CAD (cinnamyl-alcohol dehydrogenase), COMT (caffeic acid 3-O-methyltransferase), CCoAOMT (caffeoyl-CoA O-methyltransferase), CHS (chalcone synthase), 4CL (4-coumarate-CoA ligase); KAT (b-ketothiolase), CHD (cinnamoyl-CoA hydratase-dehydrogenase) BALD (benzaldehyde dehydrogenase), TE (thioesterase), BPBT (benzyl alcohol O-benzoyltransferase), F3H (flavanone 3-dioxygenase), F3′H,flavonoid-3′-hydroxylase), FNS (flavone synthase).

**Figure 7:**
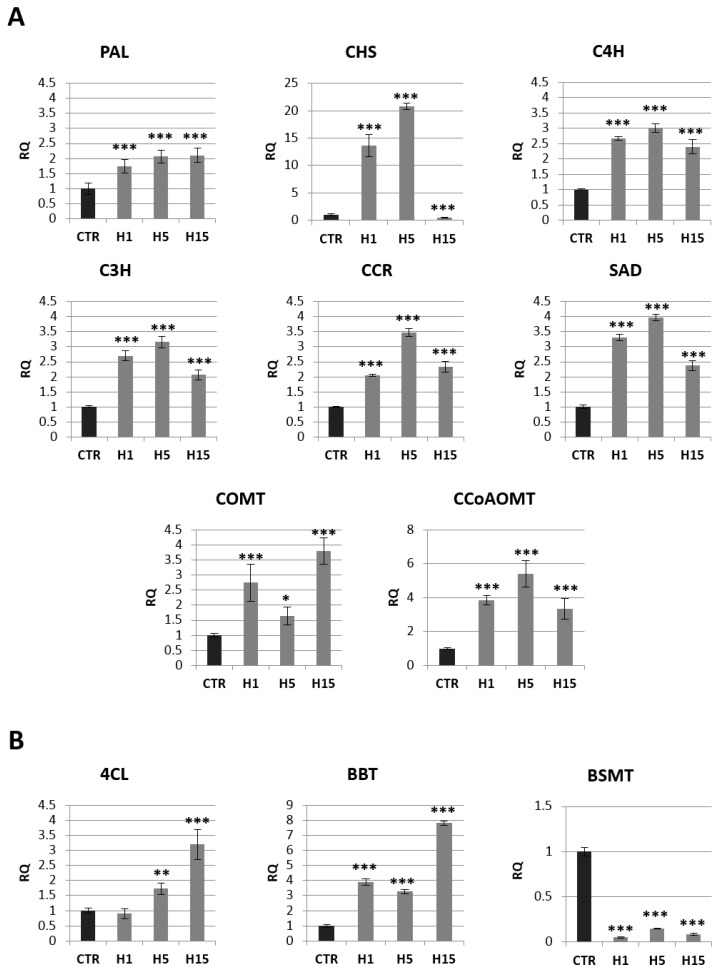
The mRNA level of selected genes involved in phenolic acids and lignin route: *PAL, CHS, C4H, C3H, CCR, COMT, CCoAOMT, SAD* (**A**), and benzoic acid route: *4CL, BBT, BSMT* (**B**) in transgenic flax plants (CTR). The mRNA level of PAL (phenylalanine ammonia lyase); CHS (chalcone synthase); C4H (cinnamic acid 4-hydroxylase); C3H (4-coumarate 3-hydroxylase); CCR (cinnamoyl-CoA reductase); SAD (sinapyl alcohol dehydrogenase); COMT (caffeic acid 3-O-methyltransferase); CCoAOMT (caffeoyl-CoA O-methyltransferase); 4CL (4-coumarate-CoA ligase); BBT (benzyl alcohol O-benzoyltransferase); BSMT (benzoic acid/salicylic acid methyltransferase) in transgenic flax in comparison with control plants (CTR) was obtained from the real-time RT-PCR analysis. *Actin* was used as a reference gene and the transcript levels were normalized to those of the control plant. RQ: relative quantity. The results are presented as mean values of three independent repeats ± SD. Statistically significant differences between the means were marked with asterisks (*—*p* < 0.05, **—*p* < 0.01, ***—*p* < 0.001).

Beta-ketothiolase is one of the enzymes involved in the synthesis of benzenoids (branch of phenylpropanoid pathway) (Figure 6). This very important secondary metabolism pathway, was analyzed in transgenic plants in more detail (Figure 6) because phenylpropanoids have many important functions in the plant due to their diverse chemical structure [26,27,28]. Analysis of the expression profile of genes involved in the metabolism of phenylpropanoid compounds in transgenic plants overexpressing *beta-ketothiolase* and control plants is shown in Figure 7A,B.

Approximately a 2-fold increase in PAL mRNA (phenylalanine ammonia lyase) was observed for all transgenic lines. The two H1 and H5 lines also showed a significant increase in *CHS* (*chalcone synthase*) gene expression relative to the control (14-fold and 21-fold, respectively). All three transgenic lines showed an increase in the mRNA level of genes involved in the synthesis of lignins and phenolic acids: *C4H* (*cinnamic acid 4-hydroxylase*), *C3H* (*4-coumarate 3-hydroxylase*), *CCR* (*cinnamoyl-CoA reductase*), *SAD* (*sinapyl alcohol dehydrogenase*), *COMT* (*caffeic acid 3-O-methyltransferase*) and *CCoAOMT* (*caffeoyl-CoA O-methyltransferase*). 

The largest increase in mRNA level was observed for CCoAOMT. The H5 line showed a 5.4-fold higher mRNA level of this gene than the control, and the H1 line showed 3.9-fold higher expression, while the H15 line showed a 3.4-fold higher level relative to non-transgenic flax. A similar expression pattern among transgenic lines was also observed for the *C4H, C3H, CCR,* and *SAD* genes. The H5 line showed a 3.0-fold increase in C4R mRNA, 3.1-fold for the *C3H* gene, 3.5-fold for the *CCR* gene, and 4.0-fold for the *SAD* gene relative to the control. The H1 line had 2.7-fold overexpression of the *C4H* and *C3H* genes, 2-fold for the *CCR* gene and 3.3-fold for the *SAD* gene relative to non-transgenic flax. The increase in mRNA level for the H15 line was 2.4-fold for C4H, 2.0-fold for C3H, 2.3-fold for CCR, and 2.4-fold for SAD. The *COMT* gene had a different expression pattern. The increase the mRNA level of this gene (a 2.7-fold increase for the H1 line, 1.6-fold for the H5 line and 3.8-fold for the H15 line compared to the control plants) was observed.

In summary, transgenic plants overexpressing *beta-ketothiolase* showed an increase in transcript levels for most of the analyzed genes involved in the lignin and phenolic acid synthesis pathway. These genes were also overexpressed in flax treated with the beta-hydroxybutyrate standard (Figure S4).

The level of mRNA for selected genes from the benzenoid pathway is shown in Figure 7B. The mRNA level of *BSMT* (*benzoic acid/salicylic acid methyltransferase*) gene that participates in the synthesis of benzenoids was reduced by 95% in H1 line, 85% in H5 line and 92% the H15 line. *4CL* (*4-coumarate-CoA ligase*) was the next analyzed gene from this pathway. The H1 line showed no change in the mRNA level of this gene, while the H5 and H15 lines showed an increase of 1.7 and 3.2-fold, respectively, in relation to the control. All three lines showed an increase in BBT (benzyl alcohol O-benzoyltransferase), the H1 line 3.9-fold, the H5 line 3.3-fold, and the H15 line 7.9-fold.

**Figure 8:**
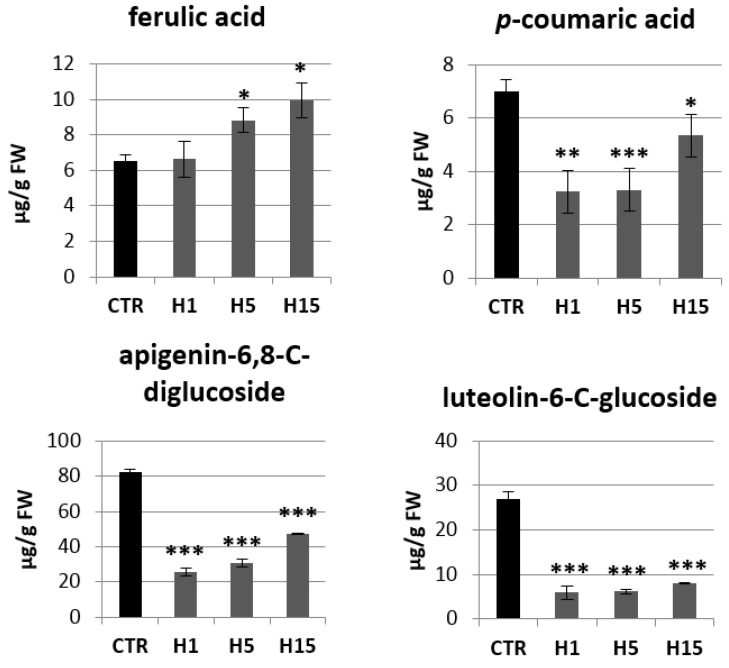
The levels of selected phenolics (ferulic acid, *p*-coumaric acid, apigenin-6,8-C-diglucoside, luteolin-6-C-glucoside) of the phenylpropanoid pathway in transgenic flax plants. Content of selected phenolic compound in 5-week-old plants (stems and leaves) from in vitro culture of transgenic flax lines H1, H5, H15, and control flax (CTR). The content of metabolites was obtained from the UPLC analysis. Calculations were performed using MassLynx software. The results are presented as mean values of three independent repeats ± SD. Statistically significant differences between the means were marked with asterisks (*—*p* < 0.05, **—*p* < 0.01, ***—*p* < 0.001).

Then, analysis of the content of phenylpropanoid compounds in transgenic and non-transgenic plants were performed (Figure 8). Transgenic plants showed a lower *p*-coumaric acid content compared to the control. The largest decrease was observed for the H1 and H5 lines, which contained about 3.25 µg/g and 3.30 µg/g *p*-coumaric acids, while the control contained 7.00 µg/g and the H15 line 5.36 µg/g. Two lines were characterized by a higher content of ferulic acid. The H5 line contained 1.3 times more ferulic acid and the H15 line 1.5 times compared to the control. All transgenic plant lines showed a significant decrease in flavonoid levels relative to the control. The H1 line contained 3.2 times less apigenin-6,8-C-diglucoside (vicenin) than the control plants, the H5 line 2.7 times less and the H15 line 1.7 times less. The content of vicenin in the H lines was in the range of 26–48 µg/g tissue, while the control plants contained about 82 µg/g. Transgenic plants also accumulated less luteolin-6-C-glucoside (isoorientin). The H1 line contained 5.93 µg/g isoorientin, the H5 line 6.19 µg/g, and the H15 line 7.99 µg/g. Wild-type flax contained 26.92 µg/g. Transgenic plants showed a higher ratio of the content of sugar derivatives of apigenin to luteolin (4.4, 5.0, 6.0 for lines H1, H5, H15, respectively) than that in the control (3.0).

## 3. Discussion

The plant growth stimulation by the degradation product (3-HB, acetyl-CoA) of symbiotic bacterial PHB, the presence of endogenous PHB in plants, and 3-HB broad activity in vertebrates, support the hypothesis that the monomer 3-HB, either synthesized de novo or derived from depolymerized PHB, is somehow involved in plant physiology. In this study, we focused on 3-HB determination, analysis of expression of genes that control its synthesis and determination of its potential function in the flax plant.

In the first instance, we concentrated on 3-HB identification. According to the retention time in GC-MS analysis and the characteristic bands for 3-HB in the IR spectra analysis, it can be determined that the samples from control and transgenic plants generated in this study contained 3-HB. Furthermore, the amount of 3-HB in the transgenic plants was higher than in the control plants. Like in microbes and vertebrates it is as yet not possible to define the origin of 3-HB in plants. It might derive both from PHB depolymerization, de novo synthesizes from acetyl-CoA or another undiscovered route. Whatever the origin, monomeric 3-HB is present in plant cells.

In order to study 3-HB function we generated transgenic plants expressing the bacterial *β-ketothiolase* gene involved in its synthesis. Selected plants showed high expression of the *β-ketothiolase* gene with increased 3-HB content from 12% to 43% of the control and acetyl-CoA quantity ranging from −11% to +23% of the control value. Interestingly, the endogenous gene was also enhanced but expression of the next gene in the pathway of 3-HB biosynthesis, *acetoacetyl-CoA reductase*, reflects metabolite (3-HB) content rather than *ketothiolase* expression pattern. Thus, we suggest that the whole pathway is controlled by the level of 3-HB metabolite. It should be added that the nucleotide sequence of bacterial *β-ketothiolase* shows little similarity to the coding sequences of endogenous *beta-ketothiolase* (35.2–46.2%), which rather precludes mutual regulation of both genes at the transcription level, underlining the suggestion of their regulation by the metabolic product.

To verify this hypothesis, control plants grown in vitro were treated with exogenously added 3-HB. The endogenous *β-ketothiolase* gene was induced by both low and high 3-HB concentrations but low concentration induction was stable up to 8 days, while high concentration induction returned to the control level after 8 days. A lot of literature data indicate that metabolic control of gene expression in plants exists and, depending on the amount of a metabolite in a cell, may have different effects on the level of gene expression [29,30].

In humans 3-HB serves as metabolic fuel in tissues, its synthesis is increased during metabolic stress conditions, it regulates neurotrophic factor expression and sugar metabolism, and it inhibits histone deacetylase. Healthy human subjects with continuous infusion of 3-HB demonstrated reduced cerebral glucose uptake and the neuroprotective effects mediated by histone deacetylase sirtuin 3 (SRT3) [13]. Therefore, in further study, we focused on the expression of genes involved in chromatin modifications (acetylation and methylation) and structural genes involved in the regulation of secondary metabolism in transgenic plants and control plants treated with exogenous metabolite. Of several analyzed genes coding for deacetylases only sirtuin 1 (SRT 1), which belongs to class III histone deacetylase (HDAC), was clearly inhibited in transgenic plants. This finding confirmed an earlier suggestion on histone deacetylase inhibition by 3-HB in human cells [10]. However, in plants, a member of HDACs class III instead of class I reported and confirmed in this work for human cells was inhibited by 3-HB. It was reported that SRTs regulate the activity of histone acetyltransferases (HATs). The HAT activity is dependent on nuclear acetyl-CoA concentrations [31,32] and the deacetylase activity of class III HDACs [33]. No changes in the mRNA level of *HAT* gene were detected in the analyzed transgenic plants. In 3-HB-treated plants, a decrease in mRNA level was observed for the analyzed histone acetyltransferases.

Literature data indicate that HDAC inhibition by 3-HB correlates with changes in gene transcription in vertebrates. For example, the *FOXO3A* and *MT2* genes, encoding oxidative stress resistance factors, were significantly activated as a result of the presence of 3-HB [10]. Also in vertebrates histone deacetylase associates with DNA methyltransferase and regulates gene expression [34].

For example, methylation of CGG trinucleotide repeats in the 5′ regulatory region of the *FMR1* (*Fragile X Mental Retardation* **1**) gene leads to its transcriptional inactivation. The reactivity of the *FMR1* gene can be obtained by using inhibitors of DNA methyltransferase, which leads to the re-accumulation of acetylated histones on the promoter [35].

The measured mRNA level of methyltransferase/demethylase in transgenic flax reveals a significant effect of 3-HB on both these processes. Of methyltransferases chromomethylase 3 (CMT3) was most highly activated and of demethylases two genes coding for decrease in DNA methylation 1 (DDM1) and DEMETR DNA glycosylase (DME) in most (two of three) transgenic lines were significantly activated. Expression levels of other genes of this class were not changed in transgenes and not affected by exogenous 3-HB. The global DNA methylation was only slightly changed in the transgene. CMT3 is recognized to modify G at CHG context and in plants requires demethylation of histone 3 at lysine 9 [36]. Mutations in the *A. thaliana* H3K9-specific methyltransferase SUVH4 and its paralogues SUVH5 and SUVH6 abolished H3K9me2 and substantially reduced CHG methylation [37]. However, in the case of flax transgene there is a slight increase of *H3K9 methyltransferase* gene expression and therefore this suggested effect does not operate.

More enigmatic is the finding that *CMT3* expression increase is accompanied by upregulation of DME and DDM1, nucleosome remodelers, which suggests that all these interact within the methylation pathway. Our finding is supported by the observation that in *A. thaliana* CHH methylation is facilitated by DDM1 and mediated by CMT2 (methyltransferase 2) [38]. Additionally, it was also reported that DDM1 participates in RNA-directed DNA methylation (RdDM) and synergizes or acts independently to silence rDNA [39,40].

**Figure 9:**
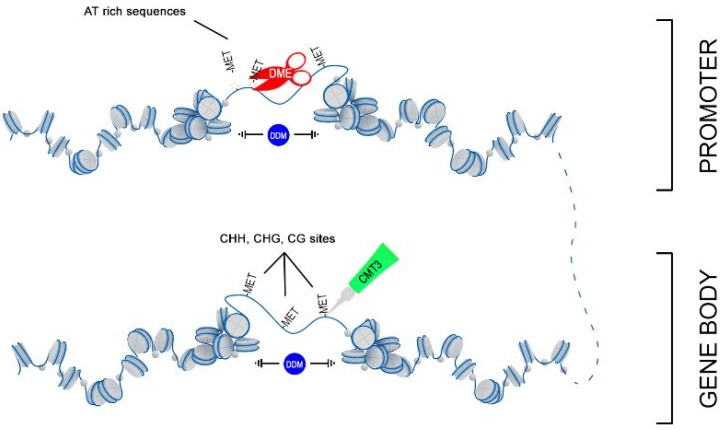
Proposed scheme of joint action of DDM1, DME, and MET3 genes in DNA remodeling.

Thus, we hypothesize that 3-HB activates expression of the chromatin remodeler DDM1, which facilitated the access of DNA methyltransferases MET3 to nucleosomes and allows remodeling of targeting chromatin (Figure 9). However, this simple picture complicates the parallel upregulation of DME, which is involved in DNA demethylation via the base-excision repair pathway. DME glycosylase enzyme mainly operates in the DNA regions enriched with AT and acts to remove 5-methylcytosine and nick the DNA backbone, followed by repair and replacement with cytosine. Recent data suggested that in *Arabidopsis* DNA demethylation mediated by DME facilitates endosperm gene imprinting and potential transgenerational epigenetic regulation of transposable element demethylation required for plant reproduction [41]. We propose that remodeled, nucleosome-depleted chromatin resulted from the combined action of DDM1 and MET3, becomes preferentially targeted to DME demethylation at the sites enriched with AT such as the promoter region and influences expression of adjacent genes.

The assumption is supported by the finding that DNA methylation regulates the structure of chromatin, leading to modulation of gene expression via changes of nucleosome positioning [42]. In addition, our own observation that the nucleosome of the *chalcone synthase* (*CHS*) gene methylated at variable motifs in the CpG island revealed higher energy and shift towards the 3’-end of the DNA also supports this suggestion [43].

To summarize, it can be concluded that greatest effect of 3-HB in flax concerns DNA methylation-demethylation rather than histone acetylation-deacetylation, but the possibility that both these processes are somehow related to 3-HB action cannot be excluded. To verify the data obtained we investigated the control plants treated with exogenously applied 3-HB. Similarly to transgenic plants, those exposed to 3-HB for 1 day (24 h) showed inhibition of *sirtuin* genes and significant activation of *CMT3* and *DDM1*, and further exposure until 8 days resulted in *CMT3* upregulation and *DDM1* downregulation; both changes were statistically significant. The *DME* gene expression did not show changes upon plant treatment with 3-HB.

This might derive from the fact that β-ketothiolase is also involved in simple phenolic biosynthesis such as benzoic acid derivatives. For example, in both bacteria and plants, phenylalanine is non-oxidatively deaminated to cinnamic acid then converted in a CoA-dependent manner to cinnamoyl-CoA and finally through β-ketothiolase and benzyl alcohol O-benzoyltransferase (BBT) via benzoyl-CoA to benzyl benzoate or by benzaldehyde dehydrogenase (BALD) and benzoic acid/salicylic acid methyltransferase (BSMT) via benzoic acid to salicylic acid [44,45,46].

In summary, stable long-lasting changes only characterize chromomethylase 3, which was upregulated upon 3-HB accumulation. We can expect that the expression changes induced by 3-HB, at least in some cases, are mediated by DNA methylation. It is known that DNA methylation is involved in plant responses to environmental conditions [47] and high reactivity of phenylpropanoid pathway genes to biotic and abiotic stressors was also observed. Notably, in transgenic flax (line H1 and H5) in particular *chalcone synthase* expression was significantly increased. We have shown recently that expression of this gene is regulated by methylation [43]. Of other phenylpropanoid pathways, the genes involved in the lignin synthesis were highly activated. This might be supported by an earlier observation that enzymes involved in the biosynthesis of flavonoid compounds and lignin occur in the form of multi-enzyme complexes in which the main regulating component is possibly CHS. Perhaps strong upregulation of *CHS* by 3-HB is sufficient to change the expression of genes involved in the lignin biosynthesis route [48]. The data from transgene analysis were mostly confirmed by the results from control flax exposed to endogenous 3-HB. We propose that 3-HB activates the expression of genes involved in the DNA methylation and through changes in the DNA methylation affects the regulation of lignin metabolism genes.

In conclusion, we detected 3-HB in flax and shown that 3-HB reveals its biological activity through activation of methylation/demethylation of the DNA pathway targeted to structural genes such as the phenylpropanoid pathway. To our best knowledge this is the first observational study suggesting the regulatory role of 3-HB in the DNA methylation pathway.

## 4. Materials and Methods

### 4.1. Plant Material

The research material was wild-type flax (*Linum usitatissimum* L. cv. NIKE) obtained from the Flax and Hemp Collection of the Institute of Natural Fibers (Poznań, Poland), and transgenic flax overexpressing *beta-ketothiolase*, as well as wild-type flax treated exogenously with the 3-HB standard. Flax plants were cultured in in vitro conditions on Murashige and Skoog (MS) basal medium (Sigma-Aldrich, St. Louis, MO, USA), supplemented with 2% sucrose (Chempur, Piekary Śląskie, Poland), pH = 5.8, solidified with 0.8% agar (Sigma-Aldrich, St. Louis, MO, USA) in the phytotron at 16 h light (22 °C), 8 h darkness (16 °C).

### 4.2. Transgenic Plant Construction and Selection

The gene encoding the bacterial *beta-ketothiolase phbA* (GenBank Database J04987.1) was cloned into the pBI 121 vector under the control of the vascular bundle-specific 14.3.3 promoter from potato (1084 bp, EMBL/GenBank Database AY070220) and fused to a plastidial targeting sequence (TPSS). The vector was introduced into the *Agrobacterium tumefaciens* bacterium (LBA4404, Invitrogen, Carlsbad, CA, USA) by electroporation.

Ten-day-old flax hypocotyls *Linum usitatissimum* L. cv. NIKE were transformed with *Agrobacterium tumefaciens* by immersing explants in bacterial suspension. Hypocotyls were incubated for 2 days in the dark at 22 °C. Then, the bacterial suspension was rinsed off using a series of rinses with sterile water and an antibiotic solution (Timentin, GlaxoSmithKline, Brentford, UK). The dried explants were transferred to the callus induction medium with a selective antibiotic and left for 10 days. After this time, calluses were transferred to shoot-inducing medium with a selective antibiotic. Emerging shoots were cut off and transferred to MS medium.

The explants were selected using PCR on a DNA template to detect a fragment of the introduced gene construct (from the promoter of the *phbA* gene) and on a cDNA template to detect a fragment of *phbA* transcript. Three transgenic flax lines were selected for further analysis.

### 4.3. Treatment of Plants with the 3-Hydroxybutyrate Standard

Four-week-old flax plants previously grown in in vitro cultures on MS medium were transferred into the MS medium supplemented with 3-HB or the control MS medium. Beta-hydroxybutyrate standard (Sigma Aldrich, Saint Louis, MO, USA) was added to the MS medium in such an amount as to obtain a final concentration of 0.2 mm and 5 mM. Treated and untreated flax plants were collected after 24 h and 8 days and stored at −80 °C for further analyses. The experiment was performed in three individual biological repeats.

### 4.4. Treatment of Human Fibroblasts with the 3-Hydroxybutyrate Standard

Normal human dermal fibroblasts (NHDF) (PromoCell, Heidelberg, Germany) were cultivated in sterile conditions in 5% CO_2_ atmosphere and 37 °C temperature. Cells were grown in DMEM medium (Lonza Group, Basel, Switzerland) containing 1.5 g/L glucose, 10% fetal calf serum, 100 U/mL penicillin and 100 μg/mL streptomycin until 90% confluence and then trypsinized (0.05% trypsin/1 mM EDTA in calcium- and magnesium-free PBS (Lonza Group, Basel, Switzerland)). Trypsin was inhibited by addition of fresh medium in 5:1 volume ratio, cells were centrifuged 800× *g* for 5 min and further plated to obtain 7 to 10 times area dilution. For the treatment, the NHDF cells were grown in cell culture dish for 24 h and then after changing the medium the 3-hydroxybutyrate at the final concentration of 0.2 mM in PBS was added. The cells were harvested after 24 h of treatment and directly used for RNA isolation (RNeasy Mini Kit, QIAGEN, Hilden, Germany, following the included protocol) and selected gene expression analysis. The experiment was performed in three individual biological repeats.

### 4.5. Determination of 3-Hydroxybutyrate Content

GC-MS analysis: 250 mg of 5-week-old green flax plants from in vitro culture, ground in liquid nitrogen, was extracted three times with acetonitrile, previously adding deuterated beta-hydroxybutyrate standard (10 µg/sample, as sodium salt dissolved in ethyl alcohol, Cayman Chemical, Ann Arbor, MI, USA) to each sample. The samples were centrifuged and the supernatant dried under a stream of nitrogen at 45 °C. The dried samples were suspended in 200 μL of BSTFA (+1% TMCS) (Sigma Aldrich, Saint Louis, MO, USA) and incubated at 70 °C for 45 min. The obtained samples were analyzed using the GC-MS technique. The analysis was performed using the Shimadzu QP2010 AOC-20i+s system (Shimadzu Corpotarion, Kyoto, Japan). Separation of compounds was achieved using a DB-5MS 30 m × 0.25 mm ID film with 0.25 µm column. The oven temperature was held at 70 °C for 3 min, then 5 °C/min rise to 110 °C and held for 0 min, 30 °C/min, increased to 300 °C and held for 10 min. The source temperature was 220 °C, the interface temperature was 260 °C and the injection temperature was 280 °C. Injection volume 1 µL, split 1: 5. Helium 6.0 with a mobile phase flow of 1.17 mL/min was used as the carrier gas. Mass spectra were obtained by electron ionization. The monitored ions in SIM mode are 117 and 121. Chromatograms and mass spectra of samples were evaluated using the GCMS solution program and the NIST14 Mass Spectral Database (National Institute of Standards and Technology, Gaithersburg, MD, USA), as well as the mass spectra of the deuterated beta-hydroxybutyrate standard.

IR analysis: Spectrum measurement was done at RT using a Bio-Rad 575C FT-IR spectrometer (Bio-Rad, Hercules, CA, USA). Data were collected in the spectral range of 50 cm^−1^ to 4000 cm^−1^ (with 2 cm^−1^ resolution). For the middle part of the range KBr (Sigma-Aldrich, Saint Louis, MO, USA) was used for sample preparation, while for the rest of the spectral range Nujol oil (Sigma-Aldrich, Saint Louis, MO, USA) was used.

### 4.6. Determination of mRNA Level

The transcript level of the investigated genes was determined using real-time PCR. The High Capacity cDNA Reverse Transcription Kit (Thermo Fisher Scientific, Waltham, MA, USA) was used to synthesize cDNA on the RNA template. The reaction was carried out according to the procedure provided by the manufacturer. Real-time PCR reactions were performed using the DyNAmo SYBR Green qPCR Kit reagent kit (Thermo Fisher Scientific, Waltham, MA, USA) in a StepOnePlus Real-Time PCR System thermocycler from Applied Biosystems (Thermo Fisher Scientific, Waltham, MA, USA). Changes in gene expression levels were presented as a relative amount with respect to the reference gene (actin). The experiment was carried out according to the manufacturer’s instructions. The annealing temperature of primers was 57 °C. The sequence of primers used for the reaction is shown in Table S1.

### 4.7. Determination of the Content of Phenylpropanoid Compounds

200 mg of green tissue (leaves, stems) from in vitro culture was used for the assay [49]. Samples were extracted three times with 1 mL of methanol (Chempur, Piekary Śląskie, Poland), centrifuged each time. The obtained supernatants were combined and dried in a vacuum evaporator, then suspended in 0.5 mL of methanol (HPLC grade, Sigma Aldrich, Saint Louis, MO, USA). To determine the content of phenylpropanoid compounds bound to the cell wall, 1 mL of 2M NaOH solution (Chempur, Piekary Śląskie, Poland) was added to the pellet remaining after extraction with methanol and subjected to alkaline hydrolysis for 24 h at 37 °C. After basic hydrolysis, the samples were centrifuged and the supernatant collected, which adjusted the pH to 3. Next, extraction was carried out three times with 1 mL of ethyl acetate (Chempur, Piekary Śląskie, Poland). Then the ethyl acetate was evaporated and the resulting precipitate was suspended in 0.5 mL of methanol (HPLC grade, Sigma Aldrich, Saint Louis, MO, USA). Initial methanol extracts and extracts formed after alkaline hydrolysis were subjected to analysis by UPLC with a diode detector (2996 PDA; Water Acquity UPLC system, Waters Corporation, Milford, MA, USA). The separation was carried out on a BEH C18 column, 2.1 × 100 mm, 1.7 μm (Waters Acquity UPLC column); 0.1% formic acid (A) and 100% acetonitrile (B) were used as eluents. The separation was carried out at a flow rate of 0.4 mL/min using the program: 0–1 min 95% A, 5% B; 2–12 min 70% A, 30%; 11–15 min 0% A, 100% B; 17 min 95% A, 5% B. Compounds were identified on the basis of retention times and the nature of the spectra, and then their quantity was determined on the basis of the appropriate standard chromatogram. The integration of the peaks was carried out at 280 nm.

### 4.8. Statistical Analysis

All experiments were independently repeated three times. The results are presented as mean values with standard deviations (SD). The statistical significance of differences between the samples was determined using the Fisher post-hoc ANOVA test. We have used Levene test to assess the equality of variances and Shapiro–Wilk test to check for normal distribution. Statistical analysis was performed using the Statistica 7 program (StatSoft, Tulsa, OK, USA).

## Supplementary Materials

Supplementary materials can be found at https://www.mdpi.com/1422-0067/21/8/2887/s1.
