# Supplementary material for: 3-Hydroxybutyrate Is Active Compound in Flax that Upregulates Genes Involved in DNA Methylation
Source: Int J Mol Sci. 2020 Apr 21;21(8):2887. doi: 10.3390/ijms21082887 (PMC7215830; doi:10.3390/ijms21082887)
Supplement: Supplementary file 1 [file ijms-21-02887-s001.zip › supplementary materials/Table S1.docx]

| **GENE** | **5’-Forward primer-3’** | **5’-Reverse primer-3’** |
| --- | --- | --- |
| Primers for the reference gene | | |
| ACTIN | CCGGTGTTATGGTTGGAAT | TGTAGAAAGTGTGATGCCAAA |
| Primers amplifying the gene fragment encoding endogenous beta-ketothiolase | | |
| Lu_bKAT | TTGCTTCCCAATTTGTCTACT | ATTGAGATGACTCCGAAACG |
| Primers amplifying the gene fragment encoding endogenous acetyl-CoA reductases | | |
| Lu_redaCoA_1 | TTCCTAGTTGGAGGAATGTTTCTTA | ATCAATCAATCAATCGCTTCAG |
| Lu_redaCoA_2 | TGTGTTTGTACGTTTCAAATTCCC | GATCGAAACTCTATCAGAGAATGC |
| Primers for genes involved in the phenylpropanoid pathway | | |
| PAL | GTTCTG TTTGAA GCCAAT GT | TGTAAG CACTCC CGTCG |
| C4H | GAGCAGGATCCGTTGTTTAAT | TCCTGTTCTTAACCTCCTCAC |
| 4CL | GCAGAAATGAAGATCGTCG | GTATGTAACCACCCTTGCT |
| C3H | CCATTGGAGTTCAAACCAGA | AGCAAGTGTCCCAACATAG |
| COMT | CTCTTGGCTTCTTACTCTGTT | TGAGGACTTTGTCCTGGTT |
| HCT | GTCGATATTCAAGCTGACCC | GTGGCGATGTACAGTTTAGT |
| CCR | ATTGGTACTGTTATGGGAAGAT | TACTTGAGGATGTGAATTGTGC |
| CC0ACMT | CGGACAAGGACAACTACAT | ACGAAGTCCCTGTAGTACCTAA |
| CAD | GGAGCATGAAGGAAACAGAG | CAACATCAACCACGAACCTA |
| SAD | CTACCTACGGAGGCTACT | GCTTGTCTAGCCCATAGAAC |
| CHS | AAATGGGGAGAATGGAAGGA | CGCACGATTCAAATAGTGAGA |
| BSMT | CGGCAACATTTATATGGCTAGTA | GATCGCTTCTTCTCCCG |
| BBT | GGTGGCGGATATGATGG | TTCCTTGTTGGTAAAGGGAATC |
| ICS | ATCGCTTGGGATAATGACC | CACAAGGCTTAATAGGAGAGTG |
| BALD | GATCGCTTCTTCTCCCG | CTCCAATCGGTTCGTGTAAT |
| SD | TGGTAGCAGTCAATCTGGTT | CCTCCAATTATGTCAGCAAG |
| CS | CGCCAATCCATGTGTTCGTA | TTTCTCTTGCAGAAGATCGG |
| ALOX | CCCATTGAACTTCATATCCG | TAGAGCTACGTGTTCGATTA |
| F3H | AGGTCGAAAGGGCAGTCTTG | GCAAGCTCCAGGGTTCAGAT |
| F3’H | TTTCGAGTTGTTGCCGTTCGG | AAATCTCCTCCATATTCA |
| FNS | ATACCCACCATGTCCAAGGC | TCCGAGTTGGTCACTACCCT |
| Primers for genes involved in epigenetic modifications | | |
| HDAC6 | GCAGATTCTTTGACGGGTGA | CCAACAGCGAGCTACATT |
| HDAC19 | GGTGCTGACTCGTTATCTG | CAGCATCGTGCAACATTC |
| SRT1 | GCTACTCAAATCAACATCCCA | TGTAACCTCAGATTTCGGTTCTA |
| SRT2 | TTCTGGATTCAAACCGATTACC | AGGCTGTGCAGCAGTAAA |
| HD2 | GCACCTGTCTCTCATAGTAG | TCTGATAGGTCATCATCATCATCG |
| HAC | AAGATTTACCGAGGACCATAC | AAGAAACAACTCGAACCACA |
| H3K9 | TGCCAAAGGTGTAAAGCTC | ATCCTTGCTTCGATTAGCC |
| GCN | TTCTGTGCGTACCTTCACT | CAGGACCTCCTCCATCT |
| CMT1 | CAGATTTCGCTCCACAGTA | AGAAATGTCCCATTGCTCTAT |
| CMT3 | AAAGGGTGCTAACTTCAGG | GACCAA ATGGTTTAGACGATGT |
| ROS | GCACTGAGAAGAAGTGCC | CTTAATGCGTGCTGCAAG |
| DME | ATGGCTACGGAGGCTACTTA | TGTTTCACCTGGTGTCCATA |
| DDM1 | GTGGTTAATATCTTTGTGGCAG | CATCCAATTTGCCAGAGTAG |
