# Supplementary material for: 3-Hydroxybutyrate Is Active Compound in Flax that Upregulates Genes Involved in DNA Methylation
Source: Int J Mol Sci. 2020 Apr 21;21(8):2887. doi: 10.3390/ijms21082887 (PMC7215830; doi:10.3390/ijms21082887)
Supplement: Supplementary file 1 [file ijms-21-02887-s001.zip › supplementary materials/Figure S3.docx]

*****

******

*****

*******

*******
